# Expression of frog virus 3 genes is impaired in mammalian cell lines

**DOI:** 10.1186/1743-422X-5-83

**Published:** 2008-07-21

**Authors:** Heather E Eaton, Julie Metcalf, Craig R Brunetti

**Affiliations:** 1 Department of Biology, Trent University, Peterborough, ON, Canada; 2 Department of Laboratory Medicine and Pathobiology, University of Toronto, Toronto, ON, Canada

## Abstract

Frog virus 3 (FV3) is a large DNA virus that is the prototypic member of the family *Iridoviridae*. To examine levels of FV3 gene expression we generated a polyclonal antibody against the FV3 protein 75L. Following a FV3 infection in fathead minnow (FHM) cells 75L was found in vesicles throughout the cytoplasm as early as 3 hours post-infection. While 75L expressed strongly in FHM cells, our findings revealed no 75L expression in mammalian cells lines despite evidence of a FV3 infection. One explanation for the lack of gene expression in mammalian cell lines may be inefficient codon usage. As a result, 75L was codon optimized and transfection of the codon optimized construct resulted in detectable expression in mammalian cells. Therefore, although FV3 can infect and replicate in mammalian cell lines, the virus may not express its full complement of genes due to inefficient codon usage in mammalian species.

## Background

*Iridoviridae *family members are large, icosahedral, double-stranded DNA viruses that are unique among eukaryotic virus genomes because they are both circularly permuted and terminally redundant [1]. The *Iridoviridae *family of viruses is comprised of five genera that can infect a variety of invertebrates (*Iridovirus, Chloriridovirus*) and ectothermic vertebrates (*Lymphocystivirus, Ranavirus, Megalocytivirus*) [2]. Specifically, *Ranaviruses *infect a variety of vertebrate hosts and have been isolated from fish, reptiles, and amphibians [3]. Frog virus 3 (FV3) is the type species of the genus *Ranavirus *and the best studied iridovirus at the molecular level. Although FV3 has not been isolated from fish, closely related viruses to FV3 including epizootic haematopoietic necrosis virus (EHNV) and Bohle virus (BIV) have both been previously isolated from a variety of fish species [4-6]. However, while FV3 is restricted to infecting a variety of amphibians and reptiles *in vivo*, fathead minnow (FHM) cells (fish) are highly susceptible to FV3 infections and are commonly used to culture the virus *in vitro *[7-9]. Therefore, FHM cells will be used to study the virus in a natural environment.

Although FV3 is unable to naturally infect any endothermic species, FV3 can infect and produce infectious virions in mammalian cell lines including human cell lines [10,11] when cultured at 30°C [9]. Mammalian cells will therefore be used to represent species that FV3 does not normally infect. Also, because of the ease with working in mammalian cell lines as compared to ectothermic cell lines, mammalian cell lines are often used to characterize FV3 genes and study virus replication. In order to further investigate FV3 infections in mammalian cell lines, we chose to examine the non-essential gene 75L, which is unique to the *Ranavirus *genus of the *Iridoviridae *family [12]. 75L, an 84 amino acid protein, has homology to cellular lipopolysaccharide-induced tumor necrosis factor-α factor (LITAF) [13] and is thought to play a role in virus-host interactions [12].

In order to determine whether FV3-75L, a non-essential gene, is expressed in mammalian cells following a FV3 infection, the mammalian cell lines BGMK (green monkey) and HeLa (human), as well as an ectothermic cell line, FHM were infected with FV3 at a multiplicity of infection (MOI) of 1. FV3 was obtained from the American Type Culture Collection (ATCC; Manassas, VA) and was propagated on FHM cells (ATCC) grown in modified Eagle's medium (MEM; Invitrogen, Burlington, ON) supplemented with 10% fetal bovine serum (FBS; HyClone, Ottawa, ON), penicillin (100 U/mL) and streptomycin (100 g/mL) at 30°C. BGMK and HeLa cells were obtained from ATCC and maintained in Dulbecco's modified Eagle's medium (DMEM; HyClone) supplemented with 7% and 10% FBS respectively, 2 mM L-glutamine, penicillin (100 U/mL), and streptomycin (100 g/mL) at 37°C with 5% CO_2_. Once infected with FV3, all cells were incubated at 30°C. At various time points post-infection, cells were fixed in 3.7% paraformaldehyde in phosphate buffer saline (PBS) for 10 minutes, and permeabilized in a 0.1% Triton X-100 solution for 4 minutes. Indirect immunofluorescence (IF) was performed [14] using either a 1/200 dilution of rabbit anti-75L antibody produced by GenScript (Piscataway, NJ), an affinity purified anti-peptide serum raised against the 75L peptide sequence CMDDKFTTLPCELED, or a 1/2000 dilution of rabbit anti-FV3 antibody (V.G. Chinchar, University of Mississippi Medical Center). The primary antibodies were detected using goat anti-rabbit FITC (Jackson ImmunoResearch Inc. West Grove, PA) and images were captured using a Leica DM SP2 confocal microscope (Leica, Wetzlar, Germany). Images were assembled using Adobe Photoshop (Adobe, San Jose, CA).

**Figure 1:**
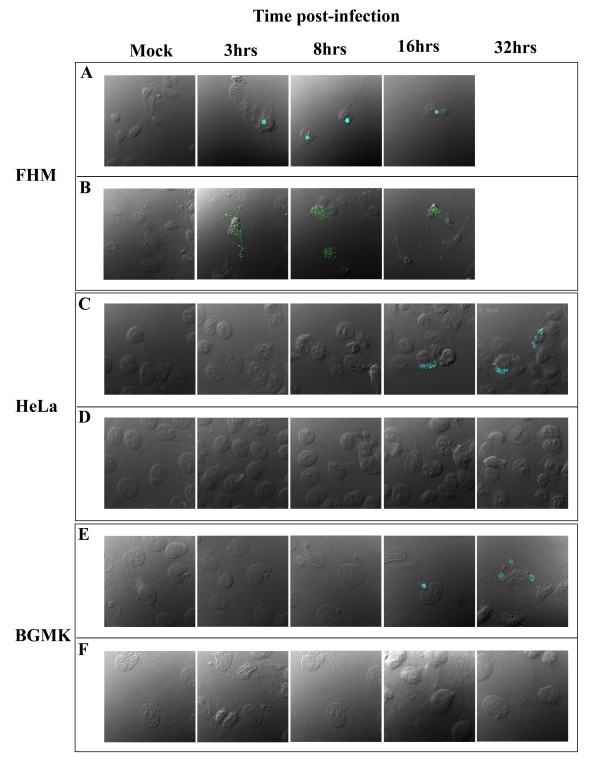
**FV3 infected BGMK and HeLa cells do not express the gene 75L**. FHM, HeLa, and BGMK cells were infected with FV3 at an MOI of 1. At 0, 3, 8, 16, and 32 hours post-infection, cells were fixed and a FV3 infection was detected using anti-FV3 antibodies (blue: A, C, E) and 75L was detected using anti-FV3-75L antibodies (green: B, D, F). No images of FHM cells at 32 hours were taken as the cells had succumbed to infection. Cells were visualized using DIC and indirect immunofluorescence images were captured on a laser scanning confocal microscope.

In FHM cells, the anti-FV3 serum was able to detect antigen as early as 3 hours post-infection (Figure 1:A). In addition, 75L expression was also detectable in FHM cells starting at 3 hours post-infection and expression increased as the infection progressed (Figure 1:B). In contrast, expression of FV3 in HeLa and BGMK cells was not detectable until 16 hours post-infection (Figure 1:C,E) and no detectable 75L expression was observed in these cell lines even as late as 32 hours post-infection (Figure 1:D,F). Therefore, although a FV3 infection was detected in all three cell lines, 75L, a non-essential gene only expressed in FHM cells, an ectothermic cell line.

**Figure 2:**
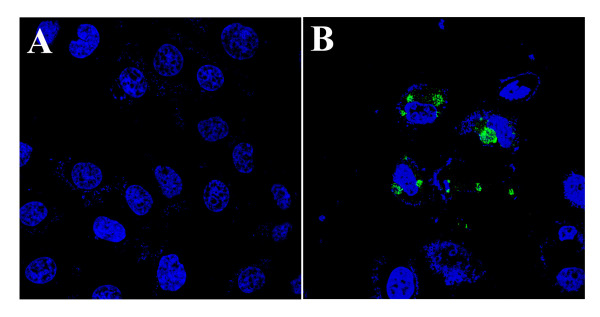
**FV3 produces infectious virions in BGMK cells**. BGMK cells were mock infected (A) or infected with FV3 at any MOI of 1 (B). 48 hours post-infection cells were harvested and virus was released. The BGMK produced virus was subsequently applied to BGMK cells and 48 hours later and the cells were fixed. FV3 was detected using anti-FV3 antibodies (green) and nucleus was visualized with ToPRO-3 (blue).

Although we demonstrated that FV3 can infect BGMK cells, we wanted to know whether FV3 produced infectious virions. BGMK cells were either mock infected or infected with FV3 at an MOI of 1 and harvested 48 hours later when cytopathic effects were seen. The cells were scraped, centrifuged for 5 minutes, and re-suspended in 100 μL of DMEM (HyClone). Following three freeze-thaws, BGMK cells were inoculated with 1 μL of the resulting suspension and were fixed 48 hours later. IF was performed using rabbit anti-FV3 antibody (V.G. Chinchar) and goat anti-rabbit FITC (Jackson ImmunoResearch Inc). Following the secondary antibody, cells were washed several times in PBS, and incubated in To-PRO-3 (Molecular Probes, Eugene, OR) for seven minutes diluted 1/10,000 in dH_2_O. The cells were washed with PBS and fluorescence was detected using a Leica DM SP2 confocal microscope (Leica, Wetzlar, Germany). Images were assembled using Adobe Photoshop (Adobe, San Jose, CA). No FV3 expression was detected in mock infected cells (Figure 2:A) while plaques (data not shown) and high levels of FV3 protein were detected after 48 hours of infection (Figure 2:B), indicating that FV3 can produce infectious virions in BGMK cells.

Since 75L was not expressed in mammalian cell lines such as BGMK and HeLa cells following a FV3 infection, we wanted to investigate whether this was a property of the 75L gene or a defect in viral expression of 75L. Therefore, we generated a C-terminal myc-tagged FV3-75L. In order to generate FV3 DNA for use in PCR, FHM cells were infected with FV3 at a MOI of 0.1. When cytopathic effects were observed, cells were harvested and re-suspended in 400 μL dH_2_O. Cells were freeze-thawed three times and an equal volume of phenol:chloroform was added and the aqueous phase was transferred to a fresh tube and 10% (v/v) 5 M sodium acetate and 20% (v/v) ethanol (100%) was added. Following a 15 minute incubation on ice, DNA was pelleted by centrifugation and 10,000 × g for 10 minutes. DNA was air dried and re-suspended in dH_2_O. A 50 μL reaction mixture containing 10 ng of DNA from virally infected cells, 1× PCR buffer (Invitrogen), 3.0 mM MgCl_2 _(Invitrogen), 0.1 mM dNTPs, 0.2 mM of FV3-75L-forward (5'-AAGCTTATTA AAGATGGACGACAAG-3') and FV3-75L-reverse (5'CTCGAGCTACAGATCTTCTTCAGAAATAAGTTTTTGTTCTAAAATTTTGTA CACAAACAC-3'), and 2.5 U of Taq DNA polymerase 5 U/μL (Invitrogen) was used to amplify FV3-75L and add a myc tag to the C terminus using the following cycling conditions: 94°C for 30 seconds, 52°C for 30 seconds, 72°C for 90 seconds for 30 cycles. The resulting product was cloned into the eukaryotic expression vector pcDNA3.1 (Invitrogen). BGMK and FHM cells were grown to 80% confluence on 22 mm coverslips in a 6-well plate. The cells were transfected with 5 μg of FV3-75L DNA using a calcium phosphate mediated transfection protocol [15]. Twenty-four hours post-transfection, the cells were fixed and processed for IF using mouse anti-myc antibody (Roche, Indianapolis, IN) to detect 75L and goat anti-mouse FITC antibodies (Jackson ImmunoResearch Inc.).

**Figure 3:**
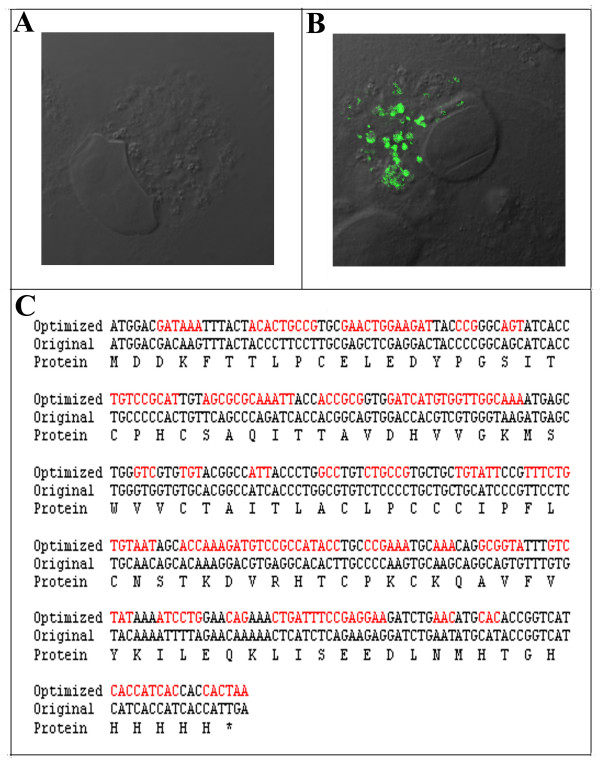
**Codon optimized 75L expresses in BGMK cells**. A FV3-75L construct tagged with a C-terminal myc tag under the control of a CMV promoter (A) or a codon optimized construct (B) was transfected into BGMK cells. Twenty-four hours post-transfection cells were fixed and indirect immunofluorescence was performed to detect 75L (anti-myc:green) and differential interference contrast (DIC) was used to visualize the cell. Images were captured on a laser scanning confocal microscope. (C) The optimized sequence is shown above the original 75L sequence, with altered nucleotides shown in red. The corresponding amino acids are shown on the bottom row and are the same for both the original and optimized sequence.

Transfection of FV3-75L in BGMK cells resulted in an absence of expression 24 hours post-transfection (Figure 3:A). This was consistent with the absence of 75L expression as a result of a FV3 infection in BGMK cells. Therefore, the lack of detectable 75L expression may be a property of the 75L gene and not a defect in viral driven gene expression. It is common for transfected viral genes to be expressed poorly in primate and mammalian cell lines. For instance, transfection of many poxvirus genes into mammalian cells results in low levels of expression [16]. Several reasons may account for this phenomenon including the use of cryptic slice sites with the pre-mRNA, mRNA instability motifs, and RNA polymerase II termination sites [16]. Another reason for poor levels of expression of viral genes may be inefficient codon usage [16-19]. The frequency that a given codon appears in a genome varies significantly between different organisms [20,21]. In order to achieve high levels of gene expression, it is important that the specific codon frequency within the gene matches that of the desired expression system. It is possible that the FV3-75L gene is optimized for expression in poikilothermic species, but not for mammalian cell lines. To determine if inefficient codon usage was responsible for the inability to detect FV3-75L in BGMK cells, a C-terminal myc-tagged construct, 75L was codon optimized (CO75L; GenScript) for *Homo sapiens *to achieve maximum expression in mammalian cell lines. Codon optimization corrects a variety of issues associated with low protein production including the replacement of infrequently used codons with those preferred by the desired host, the elimination of problematic codons, the elimination of cryptic splice sites, and the disruption of some regulatory elements that normally may result in a decrease in protein production. A comparison of the original nucleotide sequence of 75L [Gene ID 2947794] and CO75L is shown (Figure 3:C). CO75L was cloned into the eukaryotic expression vector pcDNA3.1 (Invitrogen) and transfected into BGMK cells and twenty-four hours post-transfection cells were fixed and indirect IF was used to detect 75L (mouse anti-myc and goat anti-mouse FITC conjugated antibodies). Transfection of CO75L resulted in high levels of expression compared to undetectable expression for the non-codon optimized gene (Figure 3:A,B). Expression of 75L in both BGMK and FHM cell lines revealed similar staining throughout the cytoplasm of the cell (Figure 1:B versus 3:B). The staining appears to be vesicular but may represent viral sites of replication. Therefore, the absence of 75L expression by FV3 in mammalian cells is due to inefficient codon usage.

This data demonstrates that at least one FV3 gene does not produce detectable proteins in mammalian cell lines. We believe that the lack of 75L expression is not unique to this gene as we have been unable to express a variety of FV3 genes including FV3 5R, 13R, 28R, and 29R in mammalian cell lines (data not shown). Although we have not yet shown that these genes are unable to express because of inappropriate codon usage in mammalian cell lines, the research conducted here suggests that poor codon usage is a likely reason for the lack of expression.

The consequence of codon bias in FV3 and perhaps the entire *Iridoviridae *family is that only a subset of all viral genes may be expressed in mammalian cell lines. However, essential viral genes must express in mammalian cell lines since the virus is able to infect and successfully replicate in many cell lines, including rodent, human, and simian cell lines (Figure 2) [10,11]. Although essential viral genes must be expressed, non-essential genes that are not directly involved in replication of the virus may or may not be expressed in mammalian cell lines. The possibility therefore exists that virus-host interaction may differ in mammalian cells as compared to ectothermic cell lines because the entire subset of viral genes is not expressed in mammalian cells.

Therefore, when investigating the biological properties of FV3 and perhaps other iridoviruses, it is critical that these studies be performed in ectothermic cells otherwise the entire complement of viral genes may not be expressed. In addition to the critical finding that non-essential genes may not be expressed in mammalian cells, we have also demonstrated that this expression defect can be reversed through codon optimization of the viral genes. Thus, for biochemical studies relying on the use of mammalian cell lines, codon optimization may be a solution for achieving higher levels of expression of iridovirus genes that express poorly in mammalian systems. This work has also provided a means for further characterization of the function of 75L.

## Competing interests

The authors declare that they have no competing interests.

## Authors' contributions

HEE performed the research and helped to draft the manuscript. JM helped perform the research. CRB conceived the study and participated in its design and coordination and helped draft the manuscript. All authors read and approved the final manuscript.
